# Mutation of Agr Is Associated with the Adaptation of *Staphylococcus aureus* to the Host during Chronic Osteomyelitis

**DOI:** 10.3389/fcimb.2018.00018

**Published:** 2018-02-02

**Authors:** Carlos M. Suligoy, Santiago M. Lattar, Mariángeles Noto Llana, Cintia D. González, Lucía P. Alvarez, D. Ashley Robinson, Marisa I. Gómez, Fernanda R. Buzzola, Daniel O. Sordelli

**Affiliations:** ^1^Instituto de Investigaciones en Microbiología y Parasitología Médica, University of Buenos Aires - CONICET, Buenos Aires, Argentina; ^2^Department of Microbiology and Immunology, University of Mississippi Medical Center, Jackson, MS, United States

**Keywords:** *Staphylococcus aureus*, adaptation, infection, *agr*, osteomyelitis, chronic, biofilm, capsular polysaccharide

## Abstract

Selection pressures exerted on *Staphylococcus aureus* by host factors may lead to the emergence of mutants better adapted to the evolving conditions at the infection site. This study was aimed at identifying the changes that occur in *S. aureus* exposed to the host defense mechanisms during chronic osteomyelitis and evaluating whether these changes affect the virulence of the organism. Genome assessment of two *S. aureus* isolates collected 13 months apart (HU-85a and HU-85c) from a host with chronic osteomyelitis was made by whole genome sequencing. Agr functionality was assessed by qRT-PCR. Isolates were tested in a rat model of osteomyelitis and the bacterial load (CFU/tibia) and the morphometric osteomyelitic index (OI) were determined. The ability of the isolates to trigger the release of proinflammatory cytokines was determined on macrophages in culture. Persistence of *S. aureus* within the host resulted in an *agrC* frameshift mutation that likely led to the observed phenotype. The capacity to cause bone tissue damage and trigger proinflammatory cytokines by macrophages of the *agr-*deficient, unencapsulated derivative (HU-85c) was decreased when compared with those of the isogenic CP8-capsulated parental strain (HU-85a). By comparison, no significant differences were found in the bacterial load or the OI from rats challenged with isogenic Reynolds strains [CP5, CP8, and non-typeable (NT)], indicating that lack of CP expression alone was not likely responsible for the reduced capacity to cause tissue damage in HU-85c compared with HU-85a. The production of biofilm was significantly increased in the isogenic derivative HU-85c. Lack of *agr*-dependent factors makes *S. aureus* less virulent during chronic osteomyelitis and alteration of the *agr* functionality seems to permit better adaptation of *S. aureus* to the chronically infected host.

## Introduction

*Staphylococcus aureus* is an ubiquitous opportunistic pathogen that can infect, replicate and persist in humans thus making this species a worldwide threat to public health. Although *S. aureus* may colonize mucosal surfaces of healthy humans with unnoticeable or mild clinical features, it has the invasive potential to generate diverse life threatening infections. *S. aureus* is one of the most prevalent and difficult-to-eradicate pathogens causing prosthetic device-associated osteomyelitis (Tong et al., 2015). In addition to the widespread emergence of methicillin-resistant *S. aureus* (MRSA) (Prestinaci et al., 2015), the control of *S. aureus* infections is hampered by the evolution of *S. aureus* with low-level vancomycin resistance (Howden et al., 2008).

The *S. aureus* genome carries a vast array of genes coding for virulence and immune evasion factors. Many of these genes are conserved in the *S. aureus* genome and display broad functionality with considerable redundancy (Tong et al., 2015). It is believed that initially the ability of *S. aureus* to regulate expression of certain virulence factors permits its adaptation to defined microenvironments in the infected host. Once *S. aureus* is well established at the infected tissue, and the infection becomes refractory to antibiotic treatment, certain regulatory traits may be fixed by spontaneous mutations occurring during chronic infection (Tuchscherr et al., 2010), likely due to selection pressure exerted by a vast number of yet undefined host factors. These variants are more suitable to evade immune defense mechanisms than the parental infecting wild type and are able to generate chronic infection refractory to antibiotic treatment, not necessarily associated to bacterial antibiotic resistance. A few phenotypic features such as loss of capsule (types 5 and 8) expression, loss of SSR repeats in the protein A Xr region and small colony variant (SCV) emergence have been recognized and may be considered endpoints in the short term evolution of *S. aureus* in the chronically infected host (Proctor et al., 2006; Lattar et al., 2009; Garofalo et al., 2012; Das et al., 2016). The present study was designed to investigate the main changes that occurred in *S. aureus* in a chronically infected patient persisting over a period exceeding 1 year.

## Materials and methods

### Bacterial strains

*S. aureus* clinical isolates HU-85a and HU-85c were collected as the initial isolate and 13 months later, respectively, from the same infection site (right tibia) of a 20-year-old man with chronic osteomyelitis (Hospital de Clínicas “General San Martín,” Universidad de Buenos Aires, Argentina) (Lattar et al., 2012). Species was confirmed by a species-specific PCR (Martineau et al., 1998). *S. aureus* strain Reynolds (CP5) and its isogenic derivatives Reynolds CP8 and Reynolds NT (Watts et al., 2005) were provided by Dr. Jean C. Lee (Division of Infectious Diseases, Department of Medicine, Brigham and Women's Hospital and Harvard Medical School, Boston, MA, USA). All strains were kept frozen in trypticase soy broth (TSB) with 20% glycerol at −20°C and *S. aureus* was routinely cultured at 37°C for 24 h on Columbia agar supplemented with 2% NaCl. To prepare bacterial inocula *S. aureus* was cultured on Columbia salt agar and incubated at 37°C for 24 h. Bacterial cells were harvested and suspended to the appropriate density in saline.

### The osteomyelitis rat model

Outbred Wistar adult rats weighing 250–350 g were purchased from local vendors and were kept at the vivarium of the Instituto de Investigaciones en Microbiología y Parasitología Médica (IMPaM), Buenos Aires. Animal care was in accordance with the recommendations of the guidelines set forth by: the 11 report of the BVAAWF/FRAME/RSPCA/UFAW Joint Working Group on Refinement (Hawkins et al., 2011). The animal research protocol utilized in this study was approved by the “Comité Institucional para el Uso y Cuidado de los Animales de Laboratorio,” through resolution N° 2269 issued on September 17, 2014 by the “Consejo Directivo de la Facultad de Medicina, Universidad de Buenos Aires,” Argentina. In order to produce bone infection, rats were anesthetized with ketamine/xylazine and a 5 μl suspension containing 1 × 10^6^ CFU of bacteria suspended in fibrin glue (Tissucol kit 1 ml, Baxter Argentina-AG Vienna, Austria) was injected in the left tibia as describe previously (Lattar et al., 2014). Groups of rats were sacrificed 14 weeks after intratibial challenge by exposure to CO_2_. Both left and right tibias were removed and the morphometric osteomyelitic index (OI) was assessed as detailed previously (Lattar et al., 2014). Afterwards 1 cm bone segments involving the infected zone were sectioned, crushed and homogenized in sterile mortars. Homogenates were quantitatively cultured overnight on trypticase soy agar (TSA) and the number of CFU was determined. To validate the results from the rat model experiments the correlation between the bacterial load and the OI was tested in the experiments. Phenotypic expression of α- and β-haemolysin was performed by evaluating the production of the haemolysis halo in rabbit and goat blood agar (α- and β-haemolysin, respectively). CP production was evaluated by colony immunoblot on TSA plates as described previously (Lee et al., 1990).

### Biofilm formation

Biofilm formation was quantitatively assessed according to a procedure routinely performed in our laboratory (Dotto et al., 2017). Briefly, *S. aureus* suspensions were placed in sterile 96-well polystyrene microtiter plates. After 24 h incubation at 37°C, each culture final optical density at 595 nm (named OD_G_) was measured in a microplate reader (Multiskan EX, Thermo Electron Corp., Waltham, MA, USA). The culture medium was then removed and plates were washed twice with phosphate buffered saline (PBS). The biofilms were fixed with 100% methanol, stained with 0.5% crystal violet and washed with tap water. After addition of 30% glacial acetic acid biofilm biomasses were measured by reading the optical density at 595 nm (OD_B_). The intensity of crystal violet staining was expressed relative to the final culture density measured prior to the biofilm assay (biofilm: OD_B_)/OD_G_) and termed “biofilm” in the text for the sake of clarity. Agr-deficient strain *S. aureus* SA113 (Iordanescu and Surdeanu, 1976; Periasamy et al., 2012), which is a robust biofilm producer, was included in the experiments.

### Real time quantitative reverse transcription (qRT) PCR

Bacterial RNA was extracted from *S. aureus* cultures in TSB harvested at the post-exponential phase, using Trizol Reagent® (Invitrogen Life Technologies), according to the manufacturer's protocol. RNA was subjected to DNAse treatment using a RQ1 RNAse free DNAse (Promega). cDNA synthesis was performed with an ImProm-II™ Reverse Transcriptase kit (Promega). Quantitative RT-PCR was performed using the SYBR Green PCR Master Mix (Applied Biosystems) equipment and kits. cDNA was subjected to Real time PCR using the following primer pairs: *rna*III-Fw 5′-TTC ACT GTG TCG ATA ATC CA−3′, *rna*III-Rv 5′-TGA TTT CAA TGG CAC AAG AT-3′ (Vaudaux et al., 2002); *16*S-Fw 5′-GAT CAG CAT GCT ACG GTG AA-3′ and *16*S-Rv 5′-ACC TTC CGA TAC GGC TAC CT-3′. Cycling conditions were: 95°C for 10 min followed by 45 cycles of 95°C for 10 s, 55°C for 10 s and 72°C for 15 s, and 1 cycle of 40°C for 30 s. The *16S* gene was used to normalize data. The number of copies of each sample transcript was determined with the aid of the 7500 system SDS software (Applied Biosystems). The (−Δ_CT_) value represents the difference in threshold cycle (Ct) between the target and control (*16S*) genes (Livak and Schmittgen, 2001).

### Primary cell cultures and cytokine detection

Mouse peritoneal macrophages were obtained from BALB/c mice by peritoneal lavage. Adherent cells were selected after plating the peritoneal cells in 96-well tissue culture plates. Confluent cells were stimulated 24 h later with the different bacterial strains (2–2.5 × 10^9^ CFU/well). Mouse IL-6 and TNFα were determined quantitatively at 24 h after stimulation by ELISA using specific antibody pairs (Beckton-Dickinson) as described previously (Giai et al., 2016).

### DNA extraction, whole genome sequencing, and analysis

Genomic DNA was extracted from isolates according to a standard protocol (Pitcher et al., 1989). DNA libraries were prepared using Nextera XT (Illumina, San Diego, CA, USA) and whole genome sequencing was performed using Illumina HiSeq pair-end sequencing with 500 cycles (Instituto Nacional de Tecnología Agropecuaria, Castelar, Buenos Aires, Argentina). FASTQ reads were processed with Trimmomatic (Bolger et al., 2014) to remove bases from the trailing end that fall below a PHRED score of 30. Short reads were assembled using SPAdes v3.10.1 (Bankevich et al., 2012). Contigs less than 500 bp and 30X coverage were discarded. Resulting contigs were ordered using Mauve 2.4.0 (Darling et al., 2010) and strain MRSA252 as reference. The genome was annotated using Prokka 1.12 (Seemann, 2014). Variant calling was performed using Snippy v3.2 (Seemann, 2015). The sequences reported in this paper have been deposited in the NCBI Sequence Read Archive under Bioproject number PRJNA414566.

### Statistical analysis

Groups of data were statistically compared with the Mann-Whitney test for non-parametrics. *P* < 0.05 were considered statistically significant. Multiple comparisons (three groups of rats) were performed by the Kruskal-Wallis test for non-parametrics. The correlation analysis of CFU vs. OI data was performed with the Spearman test for non-parametrics. Data from *in vitro* cytokine and biofilm production was analyzed using the Student *t*-test. The Prism GraphPad software (version 5.0) was used for all statistical analysis.

## Results

### Isolate main features

The characterization of *S. aureus* isolates HU-85a and HU-85c revealed that both were MSSA, ST188, CC1, *spa* type t189 and *agr* type I, suggesting that these longitudinally collected isolates shared a recent common ancestor at the infection site. Since HU-85a was the initial isolate and HU-85c was collected 13 months later from the same infection site, isolate HU-85a is considered to be more reflective of the “parent” isolate. Phenotypic analysis revealed that HU-85c had lost not only the capacity to express capsular polysaccharide 8 (CP8) but also α- and β-haemolysin, compared with HU-85a. These phenotypic features were stable since HU-85c did not regain CP8 or haemolysin expression upon six passages on TSA or blood agar.

### Agr functionality

**Figure 1:**
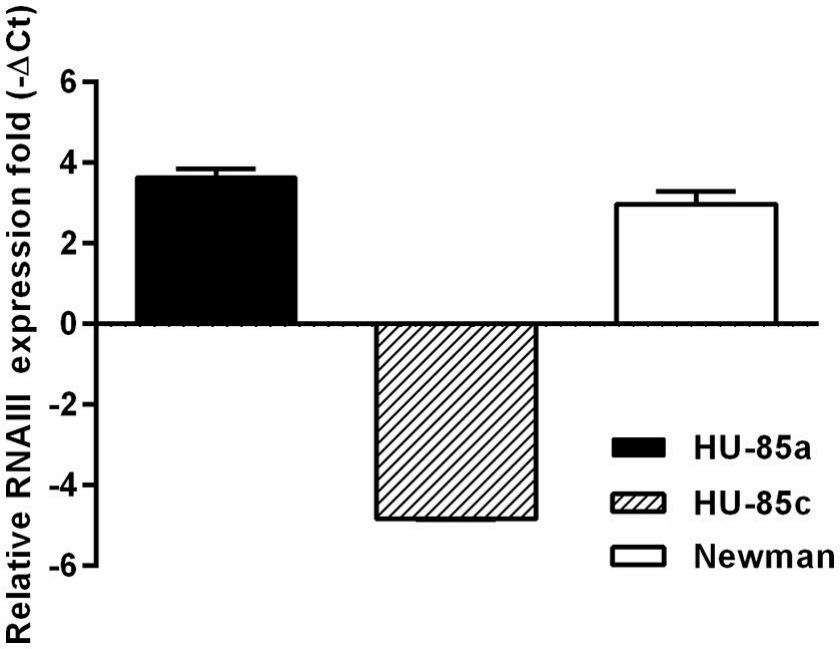
Real Time PCR of *agr* transcripts from strains HU-85a and HU-85c. Strain Newman was used as reference. Changes in gene expression are shown as normalized mean fold change −ΔCt ± SEM. Data were normalized to *16S* expression. The data represent the mean of duplicate measurements from 3 independent experiments.

To test whether HU-85c was an *agr*-deficient derivative, the expression of RNAIII from the *agr* locus was assessed by qRT-PCR. The results showed that the relative level of RNAIII expression was significantly decreased in HU-85c when compared with HU-85a (Figure 1).

### Sequence analysis

Comparative sequence analysis of HU-85a and HU-85c revealed two insertions that result in frameshifts in genes coding proteins: one in the known virulence gene regulator *agrC* and the other in the *tgt* gene encoding Queuine tRNA-ribosyltransferase, which intervenes in tRNA modification. The lesion in the *agr* locus consisted of a C insertion in position 471 of *agrC* resulting in a truncated product. In addition, there were a few other mutations, which are described in Table 1.

**Table 1:** Mutations detected by comparative sequence analysis of isolate HU-85a and its isogenic isolate HU-85c.

**LOCUS_TAG**	**Gene**	**Product**	**Type**	**Effect**	**Function**
CR496_00168	?	Hypothetical protein	del	Disruptive inframe deletion	Unknown
CR496_00524	sdrD	Serine-aspartate repeat-containing protein D	snp	Missense variant	Cell adhesion
CR496_00963	?	Hypothetical protein	snp	Synonymous variant	Unknown
CR496_00965	atl	Bifunctional autolysin	snp	Missense variant	Hydrolase
CR496_01541	tgt	Queuine tRNA-ribosyltransferase	ins	Frameshift variant	tRNA Modification
CR496_01829	pfbA	Plasmin and fibronectin-binding protein A	snp	Synonymous variant	Fibronectin binding protein
CR496_01861	mepM	Murein DD-endopeptidase MepM	snp	Missense variant	Metal binding protein
CR496_01941	agrC	Accessory gene regulator C	ins	Frameshift variant	Virulence regulator

### *S. aureus* virulence in an osteomyelitis rat model

**Figure 2:**
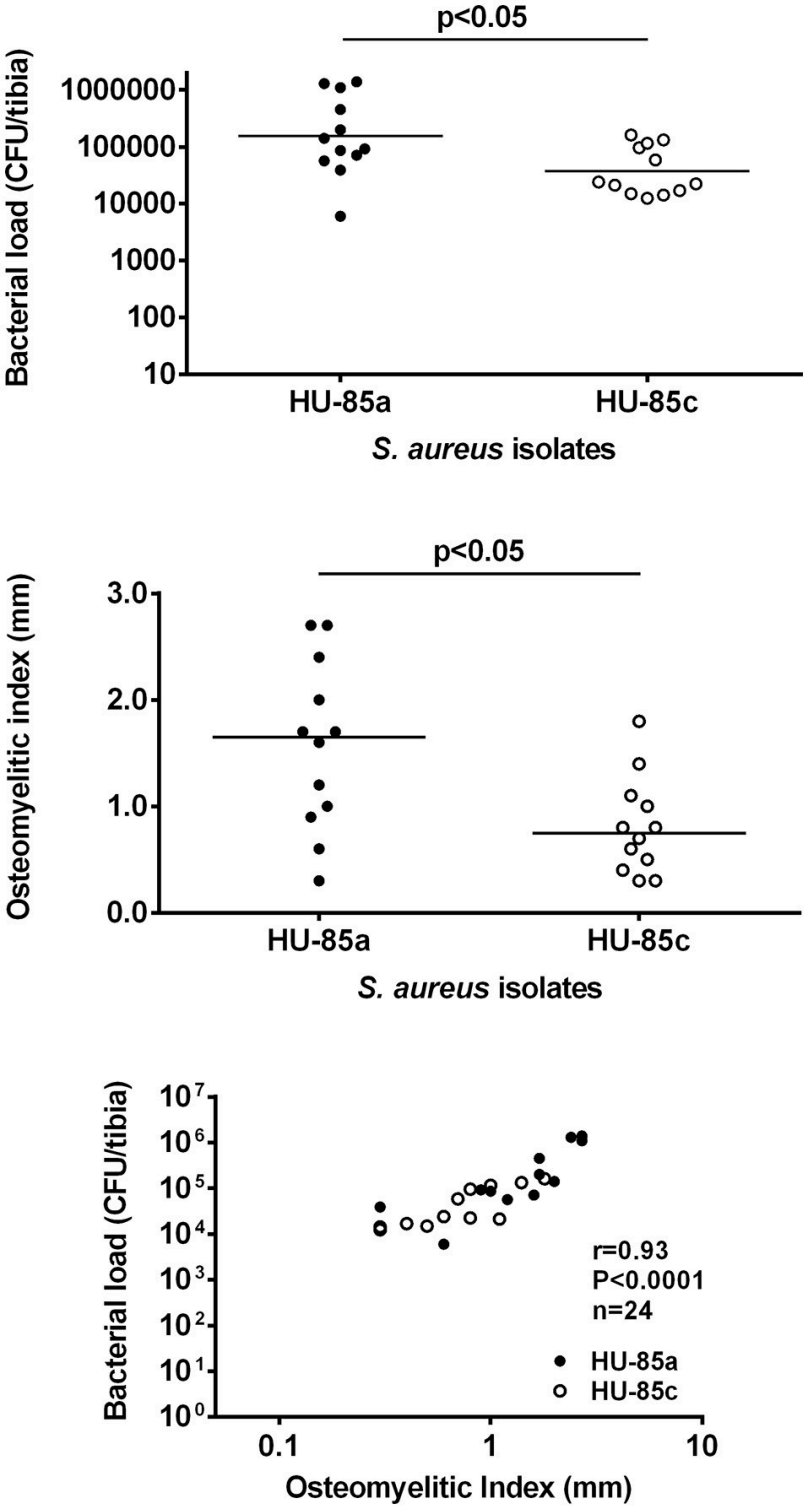
CFU/tibia (**upper**) and OI (**middle**) from rats infected with strains HU-85a and HU-85c. Bacterial loads and OI were measured 14 weeks after intratibial challenge. The sample size was *n* = 9 (HU-85a) and *n* = 12 (HU-85c), in both panels. The scattergram bars in the upper and middle panels represent the medians. The levels of significance are shown on the charts (Mann-Whitney test). The **lower** panel shows the correlation of the CFU/bone counts and the OI of all the rats included in the experiments shown in the **upper** and **middle** panels (experiment quality control).

**Figure 3:**
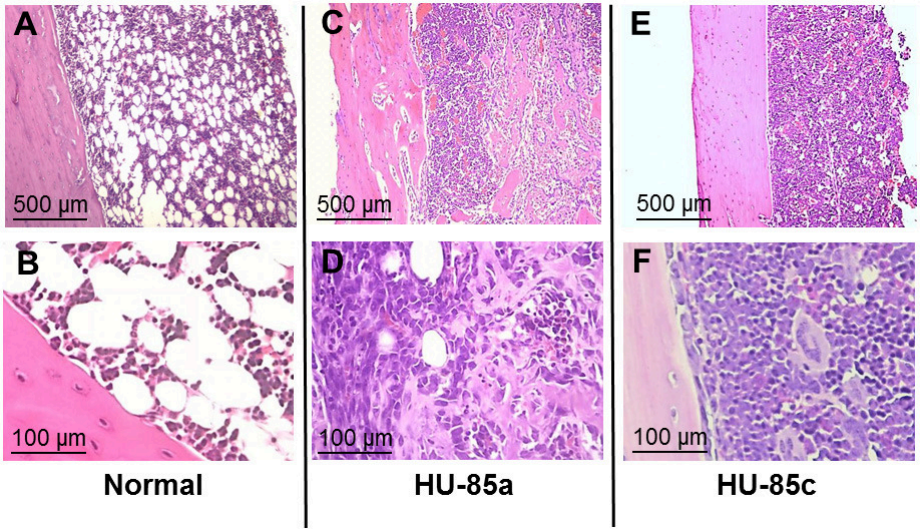
Hematoxylin-eosin-stained sections of bone after intratibial challenge with 10^6^ CFU of *S. aureus* HU-85a or HU-85c. The strain obtained earlier in the study (HU-85a; **C,D**) induced marked alterations that include bone cortex with irregular border/necrosis and bone marrow substituted by inflammatory infiltrates and invaded by trabecular structures. The strain obtained later in the study (HU-85c; **E,F**) induced less pathological changes, including significant inflammatory infiltrate composed of mononuclear cells and reduction of fat cells while the bone cortex exhibited conserved regular borders. **(A,B)** are stained sections of control, unchallenged tibias that exhibit normal bone marrow with fat tissue and cells, and a bone cortex with laminar structure.

The virulence of the two *S. aureus* isolates was evaluated in the rat model of osteomyelitis through determination of the bacterial load and the OI. Groups of rats infected with parent isolate HU-85a (*agr*+) were compared with rats infected with the derivative isolate HU-85c (*agr*-deficient). The bacterial load in the rat tibias and the OI of HU-85c by 14 weeks were significantly lower than those of HU-85a (Figure 2). No unencapsulated or non-haemolytic colonies were recovered from the bones of rats infected with HU-85a by 14 weeks after intratibial challenge. Histopathological analysis revealed that derivative isolate HU-85c caused less tissue damage when compared with the parental isolate HU-85a (Figure 3).

Whereas the loss of CP8 expression by *S. aureus* HU-85c was due to *agrC* mutation, the loss of CP is still a trait associated with chronic osteomyelitis (Lattar et al., 2009). In order to ascertain the role of CP expression loss in the osteomyelitis rat model, groups of rats were infected with *S. aureus* strain Reynolds CP5 and its isogenic CP8 and NT derivatives. The results revealed no significant differences among groups (Supplementary Figure 1). Taken together, these results indicate that the sole loss of CP5 (or CP8) expression by *S. aureus* may not be solely responsible for the reduced virulence of *S. aureus* in the rat osteomyelitis model.

**Figure 4:**
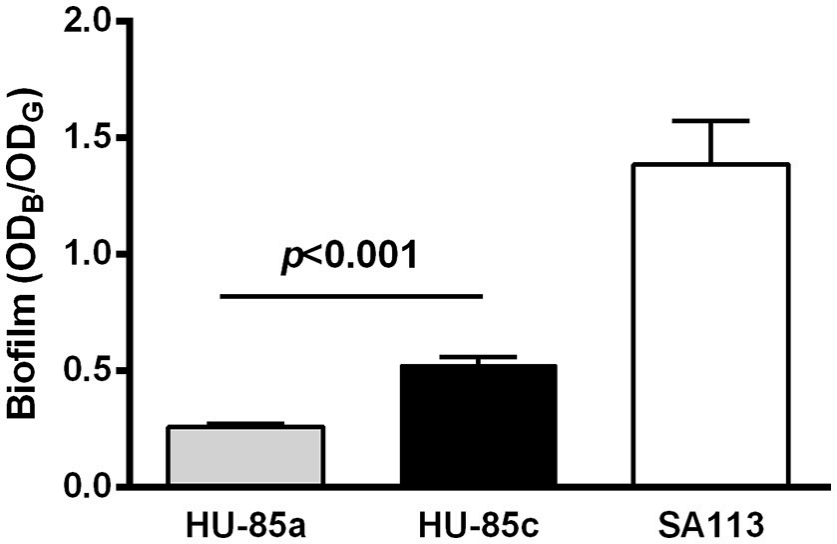
Biofilm formation by isolates HU-85a and HU-85c*. S. aureus* strain SA113 was included as reference. Each bar represents the arithmetic media ± SEM from 4 to 6 wells from 3 independent experiments. Biofilm formation values correspond to the optical density at 595 nm of crystal violet (OD_B_) measured relative to the final culture density of the bacterial growth (OD_G_) after 24 h incubation. The increase in biofilm formation by isolate HU-85c compared with HU-85a was significant (Student *t*-test).

Assessment of biofilm expression revealed that the mutation in the *agrC* resulted in a significant increase in biofilm formation by isolate HU-85c compared with isolate HU-85a (Figure 4). Reference strain *S. aureus* SA113, which is an *agr*-deficient derivative of *S. aureus* strain NCTC8325, produced the expected robust biofilm levels.

### Cytokine production by macrophages

**Figure 5:**
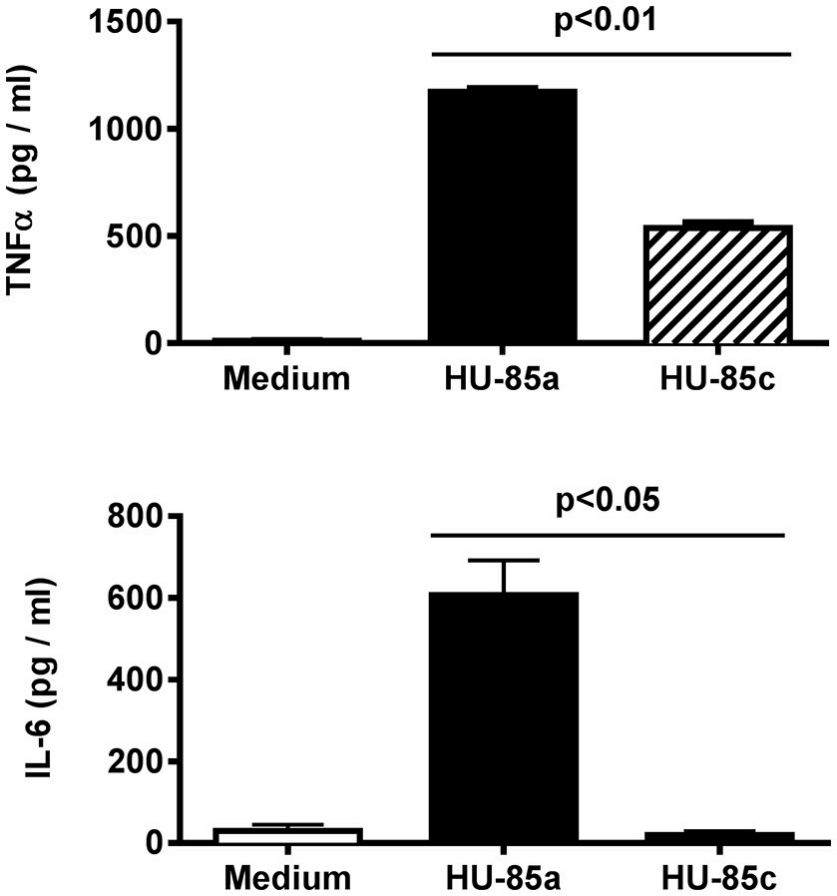
Production of TNF-α (**upper**) and IL-6 (**bottom**) by adherent mouse peritoneal macrophages stimulated with *S. aureus* HU-85a (*agr*+) or HU-85 (*agr*-deficient). Each bar represents cytokine concentrations obtained in representative experiments performed in triplicate. Comparisons of cytokine concentrations produced by stimulation with the wild-type compared with stimulation with the *agr*-deficient derivative were analyzed using the Student *t*-test.

Long exposure of *S. aureus* to the infected human bone microenvironment may select for additional phenotypic changes. We examined whether the two isolates differed in ability to induce pro-inflammatory cytokine expression. A significant reduction in TNF-α and IL-6 induction was observed in macrophages in primary culture stimulated with HU-85c (*agr*+, CP8), compared with cells stimulated with strain HU-85a (*agr*-deficient, NT) (Figure 5). These results indicate that the *S. aureus* isolate that persisted in the host with chronic osteomyelitis exhibited a reduction in its capacity to trigger an inflammatory response.

## Discussion

Genomic and phenotypic assessment of isolates HU-85a and HU-85c obtained 13 months apart from a patient with osteomyelitis revealed a frameshift mutation in the HU-85c *agrC* and a significant reduction in the RNAIII expression by HU-85c compared with HU-85a, which explain the loss of CP8, α- and β-hemolysin expression by HU-85c. Our finding agrees with those of Fischer et al., who showed in a study on *S. aureus* isolates from different sources that all non-encapsulated *S. aureus* isolated from human infections are *agr* deficient (Fischer et al., 2014). However, in *S. aureus* strains of the USA300 and USA500 backgrounds, which are not closely related to the ST188 strain studied here, the loss of capsular expression is due to mutations in the capsular genes rather than in a regulator of capsule (Boyle-Vavra et al., 2015). The *S. aureus* genome is capable of adapting to environmental changes, which can lead to significant phenotypic diversification among clinical isolates (Goerke and Wolz, 2004, 2010; Takeuchi et al., 2005). A high genetic variability of the *agr* locus has been described in *S. aureus* (Dufour et al., 2002) and attributed to balancing selection (Thomas et al., 2012). Traber and coworkers have shown that *agr*-defective variants arise by mutation during the course of infection (Traber et al., 2008). It was also reported that 9% of healthy human subjects are colonized with *agr*-defective strains (Shopsin et al., 2008) and that 22% of *S. aureus* isolates from chronically infected patients with atopic dermatitis had an *agr* mutant-like phenotype (Soong et al., 2015). In a recent study, it was shown that mutation in the *agr* locus occurs at a different rate in *S. aureus* of dissimilar clonal types (Recker et al., 2017). From the virulence standpoint, a mutation of the *agr* induces not only the lack of CP5(8) expression but also the lack of expression of secreted virulence factors (Novick, 2003), which resulted in reduced virulence in a model of septic arthritis (Blevins et al., 2002). Regulation of virulence factor expression by the *agr* system also seems to play an essential role in the adaptation of *S. aureus* to persistence at intracellular niches (Grundmeier et al., 2010). It has been shown that common single nucleotide polymorphisms that abrogate production of α-haemolysin and interfere with signaling of the *agr* reduced the virulence of contemporary hospital-associated MRSA and clinical MSSA included in *S. aureus* CC30. The authors concluded that loss of RNAIII expression, in addition to other defined mutations, made *S. aureus* less virulent to mice and provided the bacteria with suitable niche-adaptation (McGavin et al., 2012). In the present report we describe that loss of *agr* expression can occur in chronically infected patients with *S. aureus* osteomyelitis. Similarly, the reduction or total loss of *agr* functionality has been previously described in *S. aureus* from the cystic fibrosis patient lung (Goerke et al., 2000; Kahl et al., 2003). This association between persistence of *S. aureus* in the infected host and loss of *agr* expression supports the hypothesis that a non-functional *agr* may provide an advantage to *S. aureus* by defining a phenotype able to better adapt to persistence in the host.

Long-term persistence in the host leads to changes in the *S. aureus* genome. In a study on 1163 *S. aureus* genomes, Young and coworkers described one of these genome changes, i.e., the emergence of variants in protein altering genes responding to *rsp, agr* and host-derived antimicrobial peptides (Young et al., 2017). The authors further suggest that these changes emerge as a result of disease-associated, short-term, within-the-host selection pressures. Whereas in our study the factor responsible for *S. aureus* HU-85c positive selection is obscure it is interesting to note that *S. aureus* evolved from a capsulated into a non-encapsulated phenotype due to mutation in a regulator. Although diverse mechanisms account for loss of CP5(8) expression (Cocchiaro et al., 2006) so far only antibodies to CP5(8) have been pointed out as responsible for exerting selective pressure that lead to the emergence of stable NT *S. aureus* during experimental infection (Tuchscherr et al., 2008). In any event, it is speculated that adaptation of *S. aureus* for persistence at the infection site is multifactorial, as much as virulence, and that further research is needed to identify the specific host factors that exert selective pressure leading to the emergence of bacterial variants better adapted to persistence.

A major strategy displayed by *S. aureus* to support unfavorable conditions is to adopt the biofilm lifestyle, which plays a relevant role in chronic persistent infections, such as prosthetic implant associated infection (Archer et al., 2011). The importance of biofilm formation in *S. aureus* adaptation to the host to cause severe infection has been strengthened in a recent review article (Dastgheyb and Otto, 2015). In the present study it is shown that during infection mutation in the *agrC* led to significantly increased biofilm production. It is known that Agr negatively controls biofilm formation and that *agr* mutant strains of *S. aureus* produce thicker biofilms (Vuong et al., 2000). Since *agr* mutants are frequently isolated from biofilm-covered prosthetic implants, it can be assumed that robust biofilms confer an advantage to bacteria during chronic infection. Agr mutants, therefore, likely represent a dead end of infection, because they lack the capacity to disseminate within the body or establish infection in other hosts (Periasamy et al., 2012).

The progression of *S. aureus* osteomyelitis from acute into chronic may be benefited from evolution within the host from an aggressive phenotype, perhaps better adapted to cause bone damage at the start of the infection process into a less aggressive phenotype better adapted to persistence and responsible for chronic infection refractory to antibiotic treatment. The role of the evolutionary change in *S. aureus* during the progression of infection from acute into chronic has gained interest over the past few years. Expression of traits that permit adaptation of *S. aureus* to the host may initially be due to regulatory mechanisms but, in the long run, these traits may be fixed by mutation and may be considered endpoints in microevolution. One of these endpoints in *S. aureus* microevolution within the host is the stable SCV, which emerge during infection (Proctor et al., 2006; Tuchscherr et al., 2015). *In vitro* studies with host cells revealed that SCVs are phenotypes that hold low virulence, but are particularly adapted to the intracellular environment for long-term persistence (Tuchscherr et al., 2010). Interestingly, all SCV known so far are *agr* deficient thus failing to produce *agr-*regulated factors (Fraunholz and Sinha, 2012). Another endpoint of microevolution within the host is the loss of CP5(8) expression (Lattar et al., 2009). The loss of short sequence repeats (SSRs) in the protein A polymorphic region Xr is yet another endpoint, which results in the reduction of the *S. aureus* ability to trigger inflammation. In addition, the loss of SSRs was significantly higher in *S. aureus* persisting at the infection site in patients with chronic osteomyelitis or cystic fibrosis compared with patients with acute infection (Garofalo et al., 2012). In the present study, persistence of *S. aureus* in the bone and decreased ability to cause tissue damage in a rat model of osteomyelitis was associated with reduced capacity of *S. aureus* to cause inflammation, in this case through decreased triggering of proinflammatory cytokine release by macrophages. Therefore, the loss of RNAIII expression due to an *agrC* mutation and the reduction in virulence of the resulting variant makes functional impairment of *agr* another endpoint in microevolution within the host.

## Conclusion

Our study demonstrates that *S. aureus* was able to adapt to the bone during chronic infection. Stable *agr* mutants arising during human chronic osteomyelitis were less able to trigger inflammation, and to cause bone damage as ascertained in the rat model of osteomyelitis. Lack of *agr*-dependent factors turned *S. aureus* less virulent and therefore it is suggested that mutations that alter the *agr* functionality seem to permit better adaptation of *S. aureus* to infection niche, making the reduction of *agr* functionality yet another endpoint in *S. aureus* microevolution within the chronically infected host.

## Author contributions

FB, MG, and DS, conceived and designed the experiments; MN and SL, performed experiments involving the osteomyelitis rat model and data analysis; CS, DR, and DS, performed sequencing experiments and data analysis; LA and CS, performed qRT PCR experiments; MG and CG, performed cytokine experiments and data analysis; DS and CS, wrote the manuscript; FB, DR, and MG, critically revised the manuscript; DS, MG, DR, and FB, procured funding; All authors read and approved the final manuscript.

### Conflict of interest statement

The authors declare that the research was conducted in the absence of any commercial or financial relationships that could be construed as a potential conflict of interest.

## Supplementary material

Supplementary Figure 1 CFU/tibia (**upper**) and OI (**middle**) from rats infected with isogenic Reynolds CP5, Reynolds CP8 and Reynolds NT. Bacterial loads and OI were measured 14 weeks after intratibial challenge. The sample size was *n* = 14 (Reynolds CP5), *n* = 12 (Reynolds CP8), and *n* = 11 (Reynolds NT) in both panels. The scattergram bars in the upper and middle panels represent the medians. Differences among bars were not significant (Kruskal-Wallis test). The **lower** panel shows the correlation of the CFU/bone counts and the OI of all the rats included in the experiments shown in the upper and middle panels. The CFU/bone counts correlated with the OI, indicating the good quality of the results.
